# Effect of pulsatile flow perfusion on decellularization

**DOI:** 10.1186/s12938-018-0445-0

**Published:** 2018-02-01

**Authors:** Sung Min Park, Seran Yang, Se-Min Rye, Seong Wook Choi

**Affiliations:** 1 School of Medicine, Kangwon National University, Chuncheon-si, Republic of Korea; 2 Program of Mechanical and Biomedical Engineering, College of Engineering, Kangwon National University, Chuncheon-si, Republic of Korea

**Keywords:** Decellularization, Extracellular matrix, Pulsatile flow, Scaffold, Heart

## Abstract

**Background:**

Decellularized animal organs have been used as scaffolds for tissue engineering. To make a properly functioning scaffolds, the extracellular matrix (ECM) components must be preserved after decellularization. Because pulsatile flow is known to be beneficial for tissue perfusion, pulsatile perfusion of a detergent might decrease the exposure time of the tissues to the detergent used for decellularization. Using Energy Equivalent Pressure (EEP) as a pulsatility parameter, the effect of pulsatile flow in decellularization process is studied.

**Results:**

Twelve rat hearts were decellularization with 1% sodium dodecyl sulfate (SDS) solution for 2 h. They are divided into two groups, one with pulsatile perfusion (n = 6), the other with non-pulsatile perfusion (n = 6) of SDS. The initial mean perfusion pressures were same in both group. The result indicated that the EEP and the perfusion flow were increased significantly in the pulsatile group compared to the non-pulsatile group. Photographs taken during the decellularization showed more profound decellularization in the pulsatile group. The residual DNA content in the scaffolds was significantly lower in the pulsatile group. However, the level of ECM components, collagen and GAG showed no significant differences between the groups.

**Conclusions:**

Decellularization is more efficient in pulsatile flow than in non-pulsatile flow but still preserves the ECM molecules.

## Background

Immune rejection after transplantation is caused by antigens that reside in donor cells or on their surface. Removing cells from a tissue leaves the extracellular matrix (ECM), which is well tolerated, even by xenogeneic recipients [1]. The ECM consists of proteins that constitute the structural framework needed for cell adhesion and growth. Because the ECM can provide an ideal environment for cells to live and grow, using the ECM as a bioscaffold is a promising method of creating artificial organs or tissues [2].

Decellularization is a process that removes the cellular components from tissue. Among the various decellularization methods, perfusion of a detergent solution through the vascular system is a widely used procedure. The detergent breaks down cell membranes, and the intracellular components are freely washed out by the solution, leaving the ECM, which is relatively resistant to detergent extraction. Depending on the protocol, however, the ECM can be damaged by the decellularization process [3, 4]. Various ECM components which are essential for cell adhesion, growth, and migration, can be removed by decellularization. Moreover, detergent and intracellular proteolytic enzymes released by cell lysis can decompose the ECM [5]. To make a properly functioning ECM, the ECM components must be preserved as much as possible during decellularization. Because degradation of the ECM is a chemical process, shortening the decellularization time will leave the ECM bioscaffold with more intact molecules needed for recellularization [5].

Previous studies demonstrated that pulsatile blood flow is more beneficial than continuous flow for tissue perfusion [6–8]. During cardiopulmonary bypass, pulsatile perfusion resulted in increased creatinine clearance and reduced post-operative lactate level suggesting that pulsatile perfusion is beneficial in renal preservation [6]. Moreover, pulsatile flow exhibited greater benefits during and after open-heart surgery in pediatric and adult patients [7]. Therefore, we hypothesized that using pulsatile perfusion during decellularization would deliver detergent to cells more effectively, which would enhance cell lysis and, consequently, expose the ECM to detergent for a shorter time, causing is less ECM damage.

To quantify pulsatility, a parameter called Energy Equivalent Pressure (EEP) was used in this study [9]. EEP is a hemodynamic parameter that represents the hemodynamic energy of a given volume of flowing blood or liquid. The EEP can be derived from the work done in one cycle of the pulsatile flow divided by the volume of fluid moved during that cycle. In pulsatile flow, the EEP is larger than the mean pressure but if there is no pulsatility it will be equal to the mean pressure. The following formula is used to define the EEP [10]:

1 $$ {\text{EEP}} =  {{\left( {\int {\text{fp dt}} } \right)} \mathord{\left/ {\vphantom {{\left( {\int {\text{fp dt}} } \right)} {\left( {\int {\text{f dt}} } \right)}}} \right. \kern-0pt} {\left( {\int {\text{f dt}} } \right)}} $$

where f is the pump flow (ml/s), p is the arterial pressure (mmHg), and dt is the change in time at the end of flow and pressure cycles.

The concept of EEP was developed by Shepard et al. and adopted in the field of mechanical circulatory device to quantify and compare the energy of pulsatile and non-pulsatile blood flow [9–12]. Although its use in clinical practice is limited because of the difficulties in measuring continuous blood flow invasively, EEP can be obtained relatively easily from pressure and flow wave forms in experimental settings. Since the generation of pulsatile flow depends on the energy gradient rather than a pressure gradient [9–11], EEP is an excellent parameter for precise quantification of pulsatile and nonpulsatile pressure–flow waveforms [13, 14].

The purpose of this study was to determine whether pulsatile flow perfusion decreases decellularization time and preserves ECM components. To compare the pulsatility quantitatively, we used EEP as a hemodynamic energy parameter.

## Methods

**Fig. 1:**
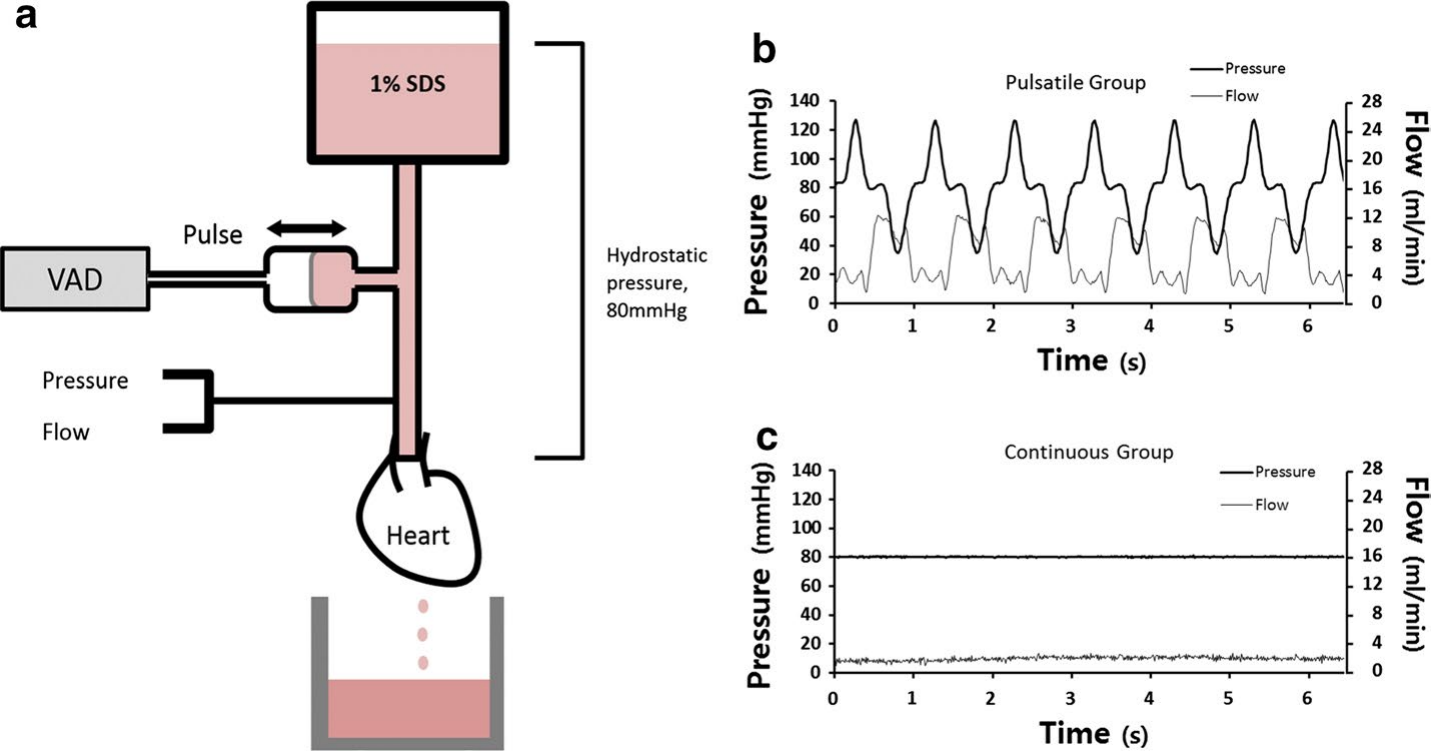
The mean pressure gradient was established by the height of the reservoir containing the solution of 1% sodium dodecyl sulfate (**a**). Pulsatile flow was generated by a pulsatile ventricular assist device. The pressure and flow of the perfusion fluid were measured simultaneously. The perfusion pressure and flow waveforms of the pulsatile group (**b**) and non-pulsatile group (**c**). The mean pressures were the same regardless of the pulsatility (**b**, **c**). *SDS* sodium dodecyl sulfate, *VAD* ventricular assist device

All animals were treated humanely, and the experiments were approved by the Institutional Animal Care and Use Committee at the Kangwon National University. Twelve Sprague–Dawley male rats (300–400 g) were anesthetized with an intra-peritoneal injection of 0.25–0.30 ml of Zoletil 50 (tiletamine/zolazepam 10 mg ml^−1^, Virbac, France,). After systemic heparinization (200 IU, Green Cross Corp, Seoul, Korea), the heart, including the aortic arch, was removed from the chest through a median sternotomy. The heart was connected to the modified isolated heart perfusion system for decellularization (Fig. 1). Depending on the type of perfusion flow, the samples were divided into two groups: the pulsatile perfusion group (n = 6) and non-pulsatile perfusion group (n = 6). After antegrade infusion with 100 ml of saline to wash out blood in the isolated rat hearts, 1% sodium dodecyl sulfate (SDS, Sigma, MO, USA) solution was perfused through the ascending aorta for 2 h. The SDS perfusion flow was continuous for the first 2 min to verify that the baseline mean pressure was 80 mmHg in both groups. Thereafter, SDS solution was perfused with pulsatile flow in the pulsatile group and continuous flow in the non-pulsatile group. After 2 h of decellularization, 200 ml of saline was infused through the aorta with non-pulsatile pressure of 80 mmHg to remove the SDS. The mid-section of the decellularized hearts were dissected for the histologic examination, and the remaining tissues were freeze-dried for analysis of cellular and ECM components. Serial photographs of the decellularizing hearts were taken to visually compare the degrees of decellularization.

To compare the effect of pulsatile perfusion on decellularization, a specialized decellularization system was made by modifying an isolated heart perfusion system (Harvard apparatus, MA, USA) (Fig. 1). The perfusion system has a SDS reservoir which was placed approximately 109 cm higher than the heart to produce a pressure gradient of 80 mmHg. This hydrostatic pressure provides the same mean pressure regardless of pulsatility. To create the pulsatile flow, a pulsatile ventricular assist device (VAD) was applied to the system. A compliance chamber was connected between the pulsatile VAD and the heart. The pneumatic pulsatile VAD, developed for a pre-clinical study, is an experimental device that can adjust stroke volume and speed. The back and forth movement of the VAD produced pulsatile perfusion of the SDS solution. The pressure and flow were simultaneously measured just above the aorta using a pressure transducer (Edwards Lifesciences, USA) and flowmeter probe (Transonic Systems Inc. USA). The pressure and flow data were recorded at baseline (without pulsatile flow in both groups), 2 min (starting pulsatile flow in one group), 1 and 2 h after decellularization began and the mean pressure and flow were calculated from 30 s data at each time period. Using the pressure and flow data, the EEP was calculated using the Eq. (1).

For the histological examination, the heart tissues were fixed in 4% paraformaldehyde for 24 h, embedded in paraffin, sectioned at 4 μm thick. The tissue slides were deparaffinized in xylene, hydrated in ethyl alcohol, and stained with hematoxylin (nucleus) and eosin (cytoplasm). After staining, the slides were dehydrated in ethyl alcohol, cleared in xylene, mounted with Canada balsam mounting solution, and viewed under an optical microscope (BX53; Olympus, Tokyo, Japan).

To measure the residual cellular components, the amount of genomic DNA was analyzed using a GeneJET Genomic DNA Purification Kit (K0721, Thermo Scientific, NY, USA). To determine the degree of ECM preservation, glycosaminoglycan (GAG) and collagen levels were measured using a Blyscan Sulfated Glycosaminoglycan Assay (Biocolor Ltd., UK) and a Sircol Collagen Assay (Biocolor Ltd., UK), respectively.

To investigate the integrity of the vascular structure, two additional rat hearts were decellularized, one under pulsatile and the other non-pulsatile condition. The decellularized hearts were perfused through the aorta with 2 ml of PBS containing 5.0 × 10^5^ of fluorescent polystyrene microspheres (10 μm, MicroProbe, MD, USA). The hearts were fixed in 4% paraformaldehyde overnight at 4 °C and sequentially placed in 10, 20 and 30% of sucrose solution. Then, the infiltrated tissues were embedded in OCT compound according general cryo-embedding protocol and cut into 20–30 μm thick sections from prepared sample. The images were taken under fluorescent microscope (BX53; Olympus, Tokyo, Japan).

Statistical analysis was performed using independent samples t test for two-group comparisons (SPSS 24, Chicago, USA). The results are shown as the mean ± SD. Statistical significance is indicated in the figure legends. A p value of less than 0.05 was considered significant.

## Results

**Fig. 2:**
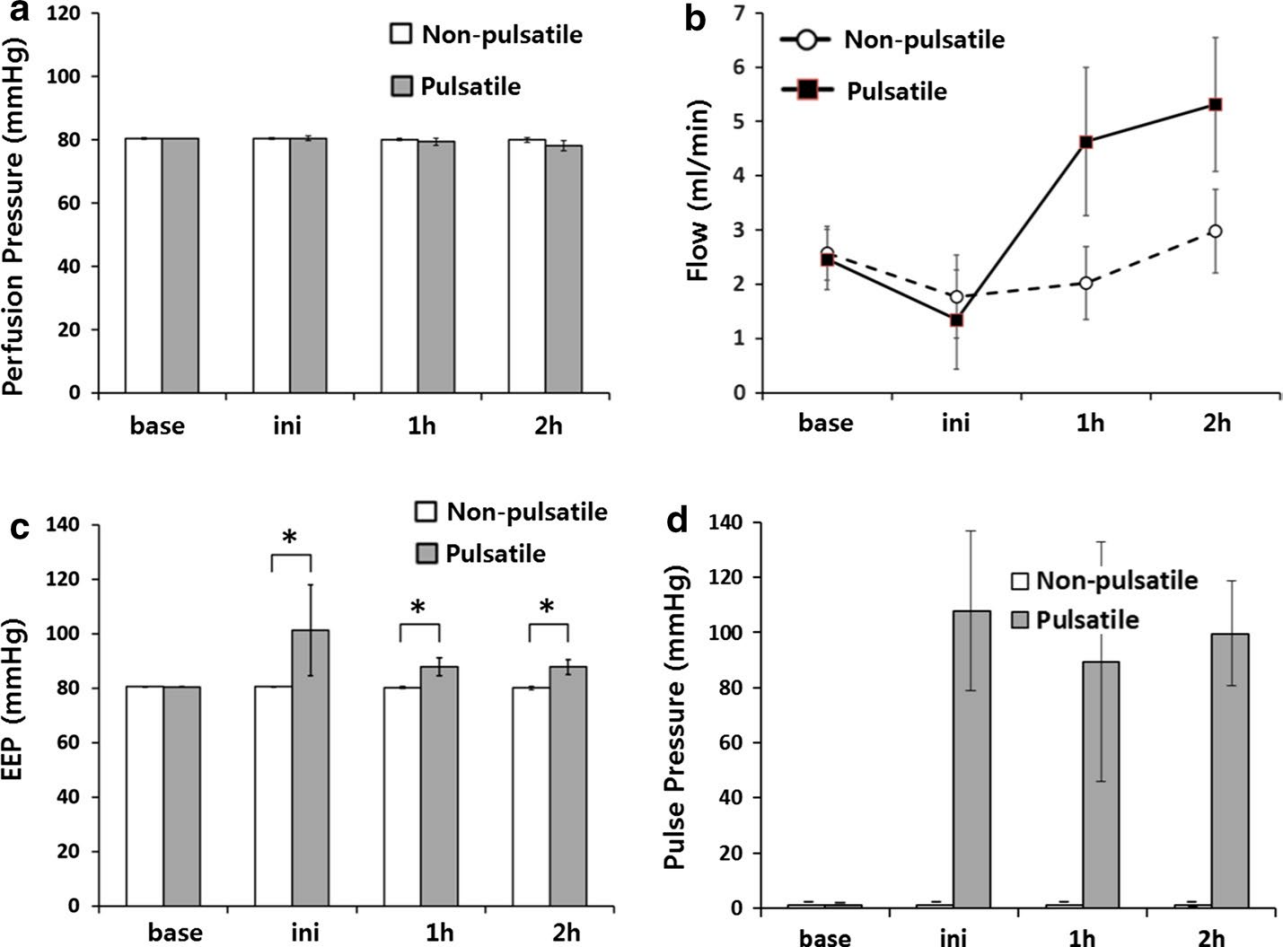
Graphs showing the mean perfusion pressure, flow and EEP of 30 s data at each specified time period. Initially, the mean perfusion pressures in both groups showed no difference (**a**). The mean perfusion flow of the SDS solution increased more rapidly in the pulsatile group (**b**). The baseline EEP (Energy Equivalent Pressure), before pulsatile flow was applied, showed no difference between the two groups. After pulsatile flow was applied, the EEP was dramatically increased (**c**). Pulse pressure shown for comparison with EEP (**d**). *p < 0.05

The weight of the rats and rat hearts before decellularization were not significantly different between the two groups. As shown in Fig. 2, the baseline mean perfusion pressure in both the pulsatile group and non-pulsatile group was 80 mmHg. After initiating pulsatile flow in the pulsatile group, the mean pressure did not change or show any significant difference from the non-pulsatile group. The mean pressures of both groups decreased slightly at 1 and 2 h after starting decellularization. The difference was slightly more prominent in the pulsatile group but not statistically significant.

The baseline EEP, taken during the initial continuous flow, was equal in both groups. The EEP increased as pulsatile perfusion began in the pulsatile group (101 ± 17 mmHg), while it remained nearly constant in the non-pulsatile group (80 ± 0.3 mmHg).

**Fig. 3:**
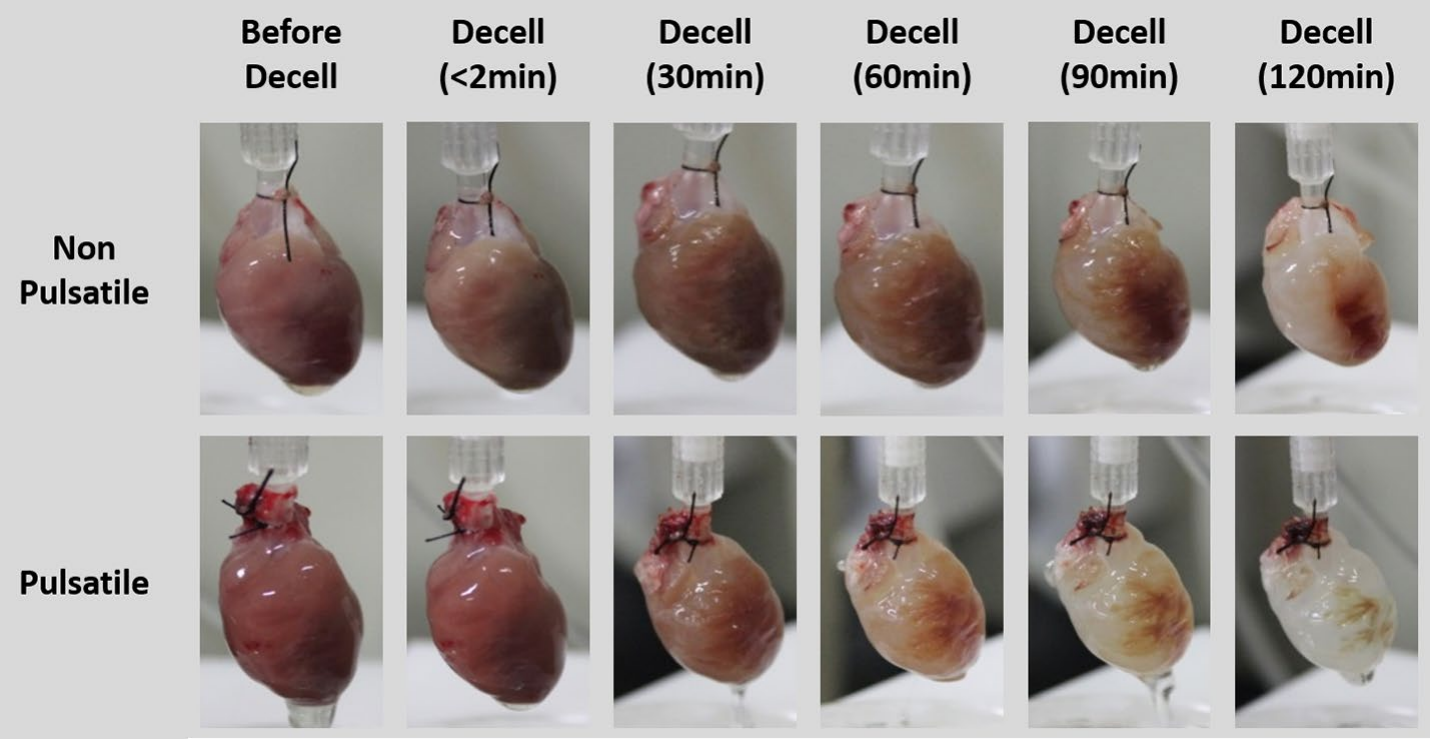
Representative images of the rat hearts during the decellularization process up to 2 h for the non-pulsatile and pulsatile group are shown. More rapid decellularization is observed in the pulsatile group

**Fig. 4:**
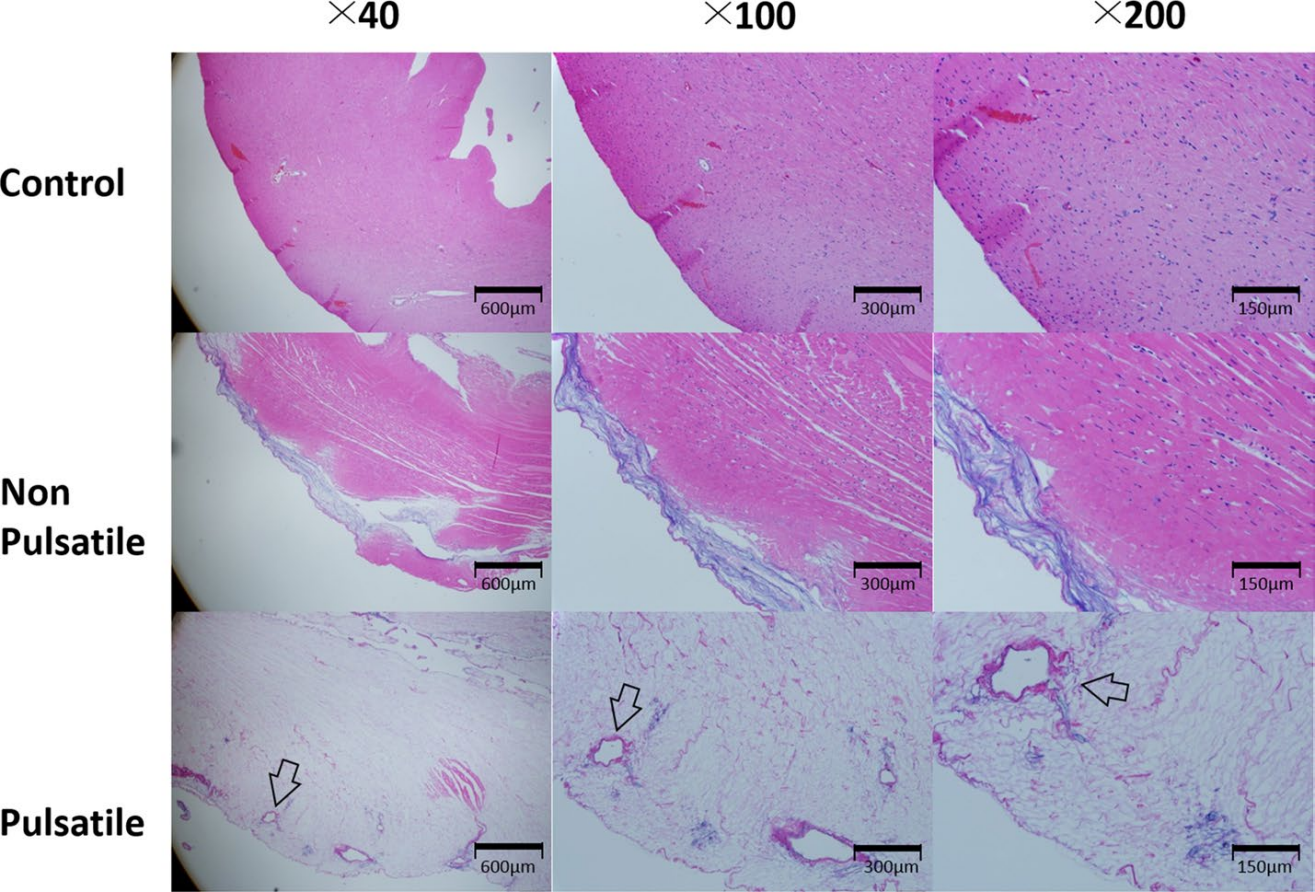
Hematoxylin and Eosin stain of the rat hearts after 2 h of decellularization showed more profound decellularization in the pulsatile group. The vascular structures are visible in the pulsatile group (arrow)

**Fig. 5:**
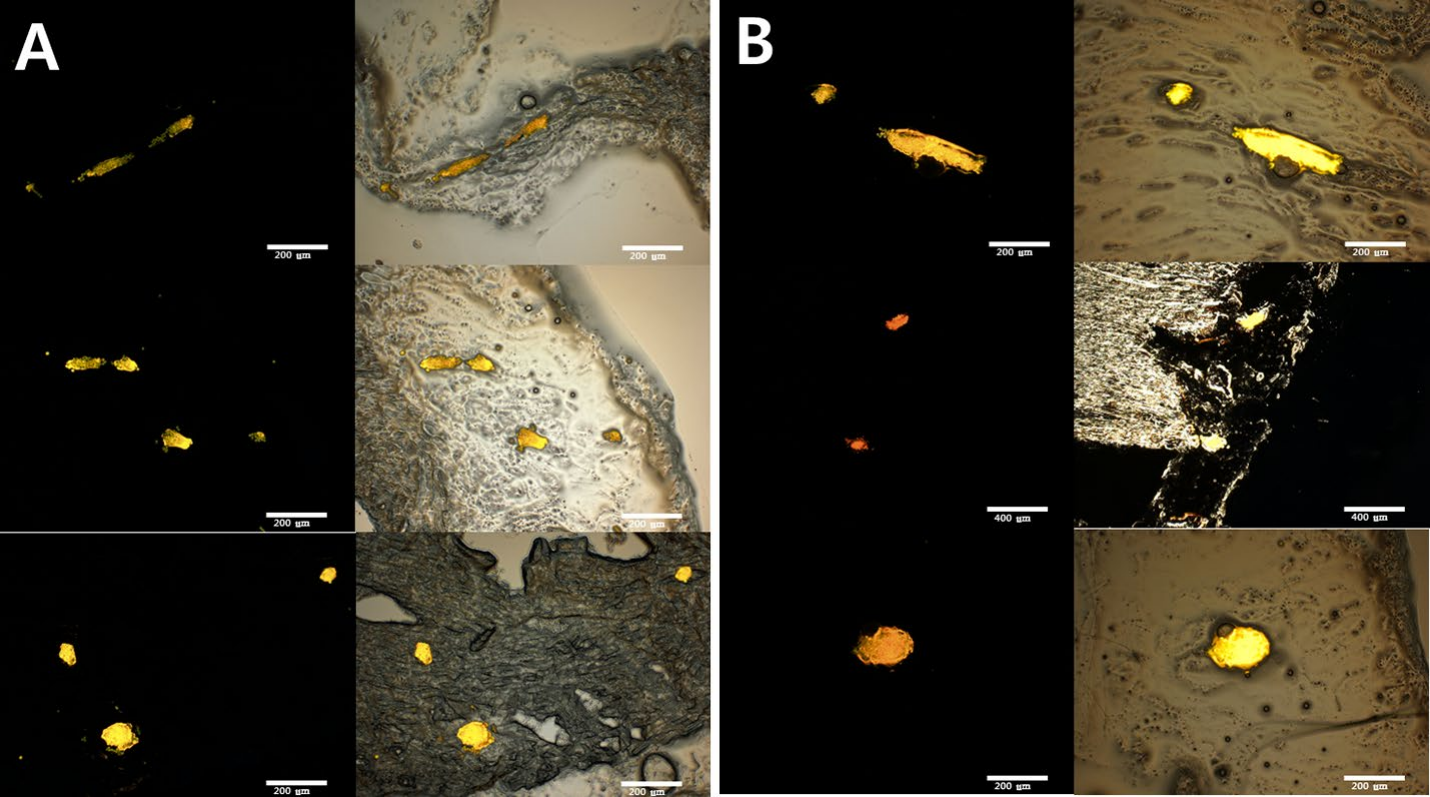
Microscopic image of fluorescent microsphere in frozen section. Fluorescent spheres form vascular shapes in the decellularized tissues of the non-pulsatile (**A**) and pulsatile group (**B**) indicating that the vascular structures are preserved (magnification ×40, ×100)

The mean perfusion flows were equal in both groups at the beginning of decellularization (non-pulsatile group 2.57 ± 0.49 ml min^−1^ versus pulsatile group 2.45 ± 0.56 ml min^−1^). The mean flows progressively increased during the process in both groups, but the increase was more rapid in the pulsatile group. After 1 h of decellularization, the mean perfusion flow was higher in the pulsatile group (non-pulsatile group 2.01 ± 0.67 ml min^−1^ versus pulsatile group 4.63 ± 1.37 ml min^−1^). Photographs taken during the process showed more profound decellularization in the pulsatile group (Fig. 3). H&E stain revealed that pulsatile perfusion more efficiently removed cellular materials than non-pulsatile perfusion, while preserving the vascular structure (Fig. 4). The frozen sections of the decellularized hearts with pulsatile and non-pulsatile perfusion showed that microspheres formed vascular shape in decellularized areas (Fig. 5).

**Fig. 6:**
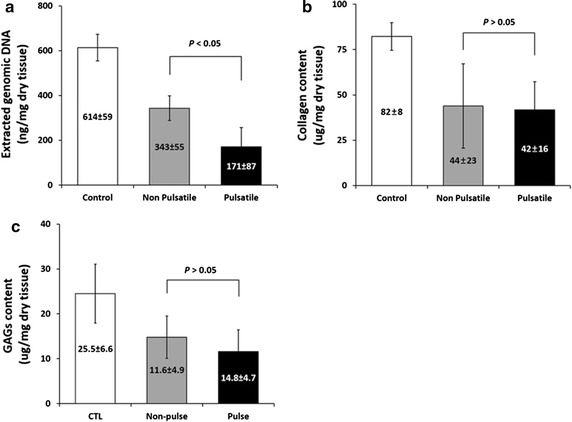
The level of cellular components, which was represented by measuring genomic DNA, is significantly lower in the pulsatile group than in the non-pulsatile group (**a**). The levels of collagen and GAG showed no significant differences between the two groups (**b**, **c**). *GAG* glycosaminoglycan

The residual DNA content in the tissue was significantly lower in the pulsatile group. However, the level of ECM components, collagen and GAG, showed no significant differences between the two groups (Fig. 6).

## Discussion

The results of our study show that pulsatile perfusion can reduce decellularization time and preserve ECM structures. After 2 h of decellularization, the levels of genomic DNA (gDNA), which represents the amount of cellular components remaining in the tissue, were lower in the pulsatile group. The levels of collagen and GAG, were not statistically different between the two groups. The photographs indicated faster decellularization, and the histologic examinations indicated more profound decellularization in the pulsatile group. The vascular structures in decellularized areas were well preserved in both groups as indicated by the aggregated shapes of the fluorescent microspheres (Fig. 5). This faster and more profound decellularization must be caused by the pulsatility of the fluid perfused.

Equivalent pressure conditions were achieved by using hydrostatic pressure to create equal mean pressures. The mean perfusion pressures of the two groups should be the same regardless of pulsatility because the height of the fluid was the same. Before starting pulsatile flow, the baseline mean pressure and flow, as well as other parameters and conditions including EEP, showed no differences between the two groups. After starting pulsatile flow, the EEP in the pulsatile group increased immediately, while the mean pressure remained mostly constant. As decellularization advanced, however, the mean perfusion pressure in the pulsatile group was slightly lower than the mean pressure in the non-pulsatile group. The higher perfusion flow of SDS in the pulsatile group resulted in a more rapid decrease of the SDS volume in the reservoir, thus decreasing the hydrostatic perfusion pressure. It is not likely that this small discrepancy adversely affects the conclusions of this study.

One of the other parameters to indicate pulsatility is pulse pressure which is the difference between the systolic and diastolic pressure [15–18]. Pulse pressure has been used clinically more widely because it is simple and easy to obtain whereas EEP requires continuous and simultaneous recording of the pressure and flow data which need invasive monitoring of the patients. However, pulse pressure does not reflect the flow component of hemodynamics (Fig. 2d). On the other hand, the EEP represents the hemodynamic energy of a given amount of flowing fluid. It is considered to be more precise in representing pulsatile component of flow and has been used to compare pulsatile and non-pulsatile flow in VAD [9–12].

The same EEP level in both groups at baseline (before pulsatile perfusion) indicates that the hemodynamic condition of the two groups were the same (Fig. 2c). The increased EEP after initiation of pulsatile flow represents the hemodynamic energy increased by pulsatility (Fig. 2c). Although EEP were high in pulsatile group, the mean perfusion pressures of the two groups showed no difference at first.

After 1 h of decellularization, higher perfusion flow was observed in the pulsatile group (Fig. 2b). The flow rate in the non-pulsatile group also increased, but the increase was slower than in the pulsatile group (Fig. 2b). The faster increase in perfusion flow observed in the pulsatile group can be explained by the higher EEP [19, 20].

During decellularization, perfusion flow usually decreases to a very low level shortly after the beginning of detergent perfusion. This phenomenon is believed to be caused by the cell debris that are initially accumulated in the vessels resulting in increased the vascular resistance during decellularization. This occlusion is relieved when the cell debris further decomposes and is removed from the vessels. Fluids with higher hemodynamic energy can overcome the higher vascular resistance. Due to the higher hemodynamic energy, as indicated by a higher EEP, pulsatile flow penetrates more easily the cell debris occluding the vessels and delivers more detergent to the tissue. The high perfusion volume of detergent observed in the pulsatile group was thus attributed to the higher hemodynamic energy originating from the pulsatility of the fluid. The higher volume of detergent resulted in more profound decellularization in the pulsatile group, as shown in the results.

There was a report in which rat livers were decellularized under oscillating and nonoscillating pressure conditions. The oscillating pressure was applied from outside of the liver and resulted in a more homogeneous distribution of detergent and improved decellularization compared to the non-oscillating condition [21]. Although not intravascular pulse, the effect of changing pressure seems to affect the distribution of detergent. Our study is first to investigate the effect of pulsatile perfusion in decellularization and to adopt EEP as a pulsatility parameter. EEP has not been used in this field, but our initial study suggests that this parameter may be well suited to the application. There are various decellularization protocols depending on the target organs and researchers’ preferences; pulsatile perfusion may be another effective method to facilitate the decellularization process.

## Conclusions

From the results, we can say that decellularization is more profound in pulsatile flow than in non-pulsatile flow but still preserves the ECM molecules and vascular structure. The difference in the level of decellularization is attributed to the higher hemodynamic energy of pulsatile perfusion, which is able to better remove cell debris from vessels, resulting in a decrease in resistance and a more rapid increase in perfusion flow. Consequently, faster decellularization is achieved when using pulsatile perfusion of detergent. Therefore, we can conclude that decellularization is more efficient with pulsatile perfusion than non-pulsatile perfusion.

## Abbreviations

EEP: Energy Equivalent Pressure
SDS: sodium dodecyl sulfate
VAD: ventricular assist device
